# Prevalence of nutritional literacy and associated factors among adult residents: a cross-sectional study from marginalized Community in Islamabad, Pakistan

**DOI:** 10.3389/fpubh.2025.1698201

**Published:** 2025-11-25

**Authors:** Abdul Momin Rizwan Ahmad, Muhammad Ajmal, Zoha Imtiaz Malik, Syed Hassan Bin Usman Shah

**Affiliations:** 1 Department of Human Nutrition and Dietetics, NUST School of Health Sciences, National University of Sciences & Technology (NUST), Sector H-12, Islamabad, Pakistan; 2 Department of Health Sciences, University of York, York, United Kingdom; 3 Department of Distance, Non-Formal & Continuing Education (DNF & CE), Faculty of Education, Allama Iqbal Open University (AIOU), Islamabad, Pakistan; 4 The Kirby Institute, University of New South Wales, Sydney, NSW, Australia

**Keywords:** nutritional literacy, adult residents, cross-sectional study, marginalized community, Pakistan

## Abstract

**Introduction:**

Nutritional literacy is one of the most important and under-explored aspects of good nutritional status especially in low- and middle-income countries.

**Objective:**

Despite its vital role in health, nutrition literacy research, particularly in low- and middle-income countries remains limited. This study aimed to assess the prevalence of nutritional literacy in marginalized settlements of Islamabad and determine the socio-demographic factors that influence nutritional literacy.

**Methods:**

A cross-sectional study was conducted among 265 adults belonging to a marginalized community of Islamabad, Pakistan. Socio-demographic data of the participants was obtained and information on nutrition literacy and health outcomes was collected using two validated tools. Data was analyzed for descriptive and inferential statistics, with significance level set at *p* < 0.05.

**Results:**

The overall prevalence of poor nutritional literacy was 89.1% in our study sample. Significant associations were found between three socio-demographic variables and nutritional literacy; gender (*p* = 0.029*), age (*p* = 0.019*), and education (*p* < 0.001*). Males and older adults were more likely to be nutritionally illiterate, while illiterate participants depicted higher odds of poor nutritional literacy. Anthropometric, vital signs, and diagnosed health conditions showed no significant associations with nutritional literacy, except skin color (*p* = 0.030), whereas cyanosis was linked to lower odds (OR = 0.203, *p* = 0.015).

**Conclusion:**

The current study reported that poor nutritional literacy is highly prevalent in marginalized communities of Islamabad and age, gender, and education, act as significant determinants. Increasing nutrition education and awareness among socially disadvantaged population groups can help promote healthier dietary practices and overall well-being.

## 1 Introduction

United Nations Educational, Scientific and Cultural Organization (UNESCO), has defined literacy as a continuum of learning which further cultivates goal attainment by empowering individuals, enabling them to become productive members of society (1). The Sustainable Development Goal (SDG) number 4, states inclusive and quality education must be available for all, and stresses on continuing lifelong learning (2). Nutritional literacy can be defined as the skills and ability to understand, analyze, and apply dietary advice and nutrition guidelines, in the presence of various interacting personal and contextual factors. It is a vital component of the food environment and exists under the ‘food and nutrition literacy’ umbrella. It equips health and nutrition professionals with the skills needed to promote nutrition knowledge among their patients, while guiding them in making informed food choices (3). It is a particularly important tool in promoting nutrition awareness and competencies among the financially disadvantaged population groups. Nutrition literacy can act as a protective shield against food insecurity and various diseases (4). Despite its significant role, and being recognized as a sub-domain of health literacy, nutrition literacy still lacks a clear definition, making it difficult to assess its role in impacting nutrition and food behaviors (5). Nutrition literacy has continued to gain recognition over the past few decades, however, research in the area still remains limited. The simultaneous rise in prevalence and awareness of chronic and non-communicable diseases (NCDs) triggered an increase in interest regarding the role of nutrition in preventing and managing such diseases. This further highlighted the importance of nutrition literacy across all age groups, although its prevalence still remains low (6). The recent prevalence of nutritional literacy in the United States of America was found to be 59% in the total population (7). An analysis of nutrition literacy in 10 Arab countries found 28% adolescent and 60% parents to be both food and nutrition illiterate. The Palestinian population reported the lowest nutrition literacy (29%) (8). A Portuguese study reported 25.6% prevalence of low nutrition literacy and a higher level in females (20.6%), as compared to males (4.5%) (9). Nutrition literacy remains particularly low in South Asia and women are majorly nutrition illiterate. The prevalence rates in Indian adolescents have been reported to be as low as 28% (10), while in Sri Lanka, 70% of reproductive age women lack basic nutrition literacy (11). Like other South Asian countries, Pakistan also reports low levels of nutrition literacy with 65.3% of adults having high basic nutritional literacy, while 34.7% reporting inadequate nutrition awareness, and 60% saying they never read food and nutrition labels (12). The nutrition literacy prevalence in adolescents is reported to be 22% and less than 15% can understand and follow a meal plan (13). College students, despite attaining formal education, are unable to comprehend, implement and critically analyze any given nutrition information, highlighting the nutrition literacy gaps among youth (14).

Nutrition literacy is effected by a number of environmental, informational, and structural factors which may either increase or decrease it. The food environment including its marketing, availability, and access, can distort the public’s understanding of food and nutrition, while also acting as barriers to its attainment, particularly in resource poor communities. Increasing amounts of conflicting nutrition information available to everyone at all times, may make it difficult to gather and apply accurate knowledge. And lastly, a lack of context specific nutrition policies or dietary guidelines may restrict the number of opportunities an individual or community may have to increase its nutrition awareness (15).

Members of marginalized communities face societal and structural exclusion due to their race, gender, ethnicity, or socio-economic status. This ultimately impacts their access to education, healthcare and quality of life. Their inequitable social status puts them at greater risk for several health conditions and compromised nutritional status (16). In terms of nutrition, the South Asian marginalized communities often report increased intake of processed diets due to the nutritional transition. Furthermore, consumption of tobacco and alcohol is prevalent, putting them at a high risk for several NCDs (17). Women report a higher disease burden with 66% hypertensive, 8.9–16% diabetic, 33.8–77% obese, and 25% diagnosed with metabolic syndrome (18). Around 43% women living in illegal settlements in Islamabad, do not have any formal education (19), whereas 48% of the population residing in informal settlements in Pakistan, lacks access to safe water, food, and health services (20). As Pakistan continues to grapple with the increasing prevalence of NCDs and chronic diseases, the growing epidemic of nutrition literacy has amplified poor nutrition and health outcomes (21). The issue is many folds greater in marginalized communities, where lack of resources make it difficult to acquire, prepare, and store nutrient rich foods. This adds to their nutrition issues, making them vulnerable to various health risks (22).

While nutrition literacy is gaining attention worldwide as key determinant of dietary behaviors and outcomes, its assessment instruments have certain contextual limitations, thereby making them unfavorable to be applied in LMICs, such as Pakistan. The most extensively administered nutrition literacy assessment tool is the Nutrition Literacy Assessment Instrument (NLit). It was developed and validated among adults living in the United States, and despite the questionnaire exhibiting high reliability and validity, it was primarily designed to capture the nutrition related knowledge in individuals living in high income countries (HICs) (23). The tool focuses mainly on supermarket skills, food label reading and numeracy, which may not be applicable while assessing nutrition literacy in a marginalized community in Pakistan. Similarly, the Newest Vital Sign (NVS), was developed to capture the health literacy among respondents, by asking them to interpret food labels. However, the tool heavily relies on food label understanding and knowledge, which again may not be able to accurately capture the broader determinants of nutrition literacy (24). Therefore, we developed a contextually relevant instrument that captures all aspects of nutrition literacy, specific to a Pakistani marginalized community.

Determining the prevalence of nutritional literacy and its relationship with various socio-demographic can aid the development of effective and culturally contextualized nutritional literacy public health interventions focused on the residents of marginalized communities. These programs can then be up scaled and adapted locally to increase nutrition literacy among all Pakistani marginalized communities, to improve their nutrition and health outcomes. The aim of our study was therefore, to determine the prevalence of nutrition literacy among marginalized community residents and to determine how various socio-demographic, health related factors may associate with nutrition literacy levels.

## 2 Materials and methods

### 2.1 Study design

The current research employed a cross-sectional survey design.

### 2.2 Study settings and population

The research was conducted in the marginalized communities of Islamabad Capital Territory (ICT), Pakistan. The study participants included both male and female adults aged 18 years and above living in these marginalized communities.

### 2.3 Sampling technique

The study used purposive sampling to collect data from 2 out of the 8 marginalized communities of ICT (Tent Colony of G-7/1 and Essa Nagri of I-9/1).

### 2.4 Sample size calculation

The sample size for the study was calculated using WHO’s sample size calculator. We assumed:

80% poor literacy prevalence (as calculated from pilot study).

5% margin of error.

95% confidence interval.

10% non-response rate.

The total sample size was calculated to be 271 participants, however, 265 took part in the study.

### 2.5 Data collection tool

A data collection instrument for determining nutrition literacy level among the study population was developed after a comprehensive literature review (Supplementary file A). The final version of the nutrition literacy instrument consisted of 14 statements, each with yes, no, and do not know as a response option. The ‘I do not know’ option was included to discourage guessing and to allow participants to differentiate between uncertainty and actual incorrect beliefs. When scoring, correct responses were coded as 1, while both incorrect and I do not know responses were coded 0. This is consistent with a similar study assessing nutrition and food literacy among multiple sclerosis patients (25).

For nutrition literacy, based on the percentage of correct responses, participants were categorized into three levels:

- Poor Nutrition Literacy: Score <60% (i.e., 0 to 8 correct answers)
- Moderate Nutrition Literacy: Score between 60 and 80% (i.e., 9 to 11 correct answers)
- Good Nutrition Literacy: Score >80% (i.e., 12 to 14 correct answers)

The cut-offs for the nutrition literacy scores were based on the Bloom’s criteria, where <60% indicates poor literacy, 60–80% indicates moderate literacy, and >80% indicates good literacy. The Bloom’s criteria has been consistently used in knowledge, attitude, and practices (KAP) studies (26).

The tool was first composed in the English language and then underwent Urdu translation by two Urdu school teachers. It was then translated back into English to preserve the original content of the tools.

The validity of the data collection tools was determined using the Content validity index (CVI). Six subject experts evaluated the instrument for simplicity, relevance, and clarity, using a 4-point Likert scale. The tables for CVI scores are given as Supplementary files B Table S1–S3. CVI scores of ≥ 0.78 indicated excellent content validity, whereas any items with scores less than 0.78 were removed (27). The content validity ratio (CVR) was also calculated for each item in the instrument as shown in Table 1. The same cut off of 0.78 was applied for CVR as well. Based upon these CVI and CVR scores, 1 item was removed from the nutrition literacy scale, and hence, the final tool therefore contained 14 statements.

**Table 1:** Content validity ratio for nutrition literacy instrument.

Item	Description	Exp. 1	Exp. 2	Exp. 3	Exp. 4	Exp. 5	Exp. 6	CVR
1	I know that eating chapati/ rice/ cereal is important because it gives me energy.	E	E	E	E	E	E	1.00
2	I understand that eating meat/eggs/lentils is essential for my health.	E	E	E	E	E	E	1.00
3	I realize that adequate consumption of milk and/or milk products is associated with strong bones.	E	E	E	E	E	E	1.00
4	I know that regular consumption of fresh fruits and vegetables can prevent me from several diseases.	E	E	E	E	E	E	1.00
5	I understand that too much consumption of salt and sugar is bad for health.	E	E	E	E	E	E	1.00
6	I know that eating meat is required for maintaining appropriate iron levels in blood.	E	E	E	E	E	E	1.00
7	I understand that sunlight is necessary to get adequate vitamin D.	E	E	E	E	E	E	1.00
8	I know that exclusive breastfeeding is the best choice for newborns.	E	E	E	E	E	E	1.00
9	I realize that mental stress negatively affects diet.	E	E	E	E	E	E	1.00
10	I know that a healthy diet positively influences sleep.	E	E	E	E	E	E	1.00
11	I am aware that washing hands with soap before handling food prevents several diseases.	E	E	E	E	E	E	1.00
12	I can read a food label.	E	E	E	E	E	E	1.00
13	I realize that one should always consult a healthcare provider before taking any nutritional supplement.	E	E	E	E	E	E	1.00
14	I can search for authentic nutrition related information on the internet.	E	E	E	E	E	E	1.00

E, Essential; U, Useful but not Essential; NE, Not Essential.

Internal consistency for the tool was assessed using Cronbach’s alpha applied through the SPSS statistical software, version 27.0. Cronbach’s alpha for the developed nutrition literacy tool was 0.714 (28).

The scoring for the instrument was done as 1 for yes, 0 for no or do not know. The total nutrition literacy score ranged from 0 to 14.

### 2.6 Pilot study

The pilot study was conducted on 30 participants, with 15 participants from each of the two marginalized communities. The purpose of the pilot study was to determine the feasibility of the developed data collection instruments. The researcher collected data for the pilot study himself.

### 2.7 Data collection for the study

To collect data for the actual study, two trained nutritionists and two phlebotomists were a part of the data collection team, with being male and one female. This was done purposively to make sure participants of both genders were comfortable during data collection. Data collection forms were administered in Urdu. Literate participants filled their own data collection instruments, while those who could not read or write were helped by the data collectors.

### 2.8 Study outcome variables

Data was collected on socio-demographic variables; age, gender, education status, employment status, marital status, and family structure. Additionally, health assessment data was collected on body mass index (BMI), waist circumference (WC), skin color, nausea/vomiting, and stool frequency. Vital signs including body temperature, blood pressure, pulse/heart rate, respiratory rate, and blood sugar random were also assessed as per their standard protocols. Data on nutrition literacy was obtained using the developed instrument.

### 2.9 Data analysis

The IBM SPSS version 27.0 was used to enter and analyze data. Mean and standard deviation were reported for quantitative data, whereas qualitative data was reported as percentages and frequencies. The association between qualitative variables was determined using chi-square or fisher exact test, while odds were determined via binary logistic regression. *p*-value of <0.05 was considered to be statistically significant.

## 3 Results

### 3.1 Socio-demographic variables

Out of the 265 study participants, 149 (56.2%) were males whereas 16 (43.8%) were females as shown in Table 2. Most of the study participants were below 30 years of age (30.6%), and only 10 participants were above 45 years of age (38.5%). Majority of the participants (55.5%) had no formal education, and only 5.3% had graduated or possessed higher education. Of the total 265 participants, majority were unemployed (17%), whereas welder and lecturer (0.4%), and postman and waiter (0.8%), were the least reported occupations. About 69.4% were married, and 89.8% were living in a joint family structure.

**Table 2:** Socio-demographic profile of the study population.

Variable	Frequency (%)
Gender	
Male	149 (56.2)
Female	116 (43.8)
Age group	
≤30 years	81 (30.6)
31–45 years	82 (30.9)
>45 years	102 (38.5)
Education level	
No formal education	147 (55.5)
Primary	58 (21.9)
Middle	14 (05.3)
Matric	18 (06.8)
Intermediate	14 (05.3)
Graduation or above	14 (05.3)
Employment status	
Un-employed	45 (17)
Private job	170 (64.2)
Govt. job	12 (04.5)
Own business	38 (14.3)
Marital status	
Single	81 (30.6)
Married	184 (69.4)
Family structure	
Nuclear	27 (10.2)
Joint	238 (89.8)

### 3.2 Prevalence of nutrition literacy

**Figure 1:**
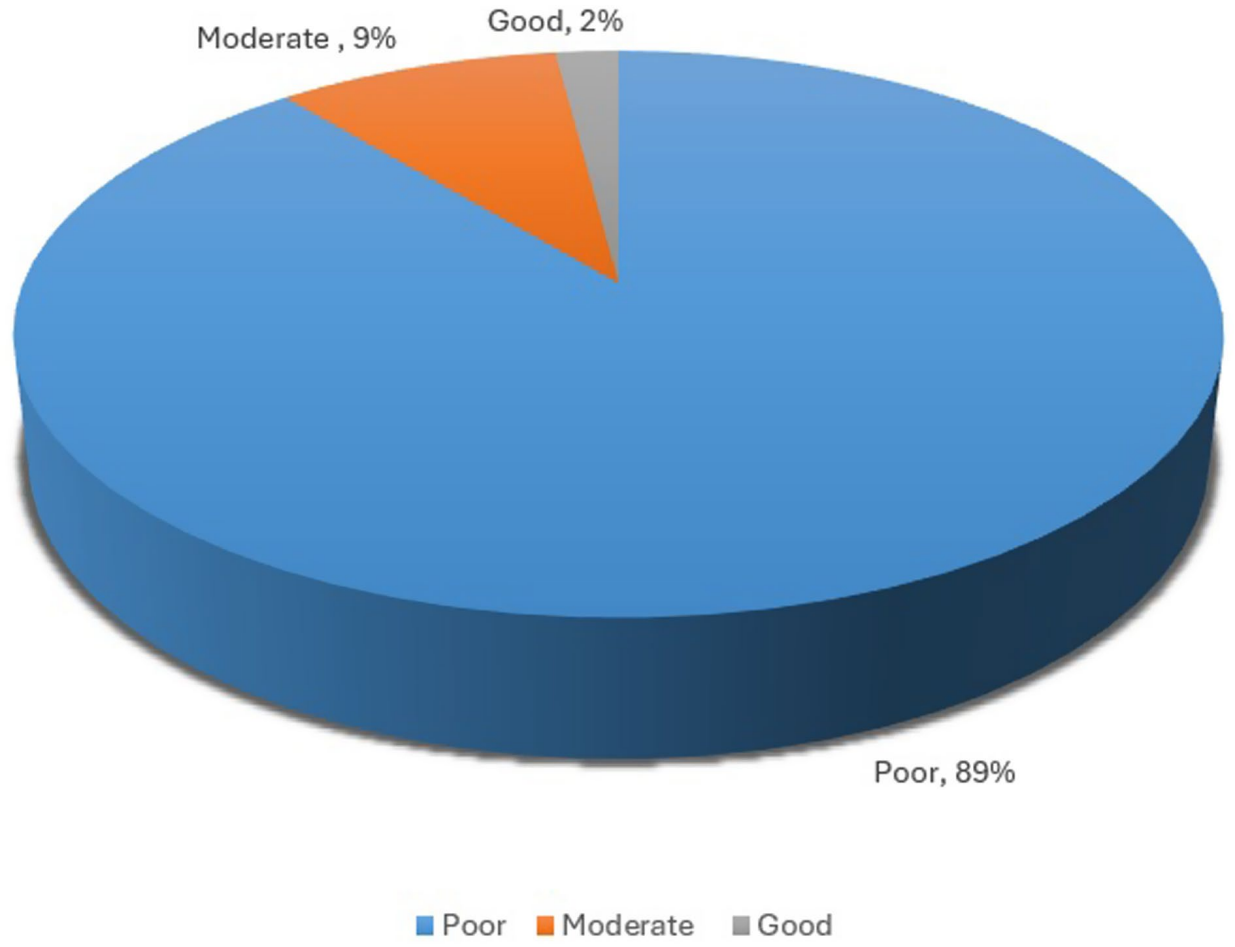
The pie chart displays the percentage of participants across different levels of nutritional literacy. Majority (89%) of the participants fell into the poor nutrition literacy category.

Out of the total 265 respondents, the majority (89.1%) had poor nutritional literacy, and only 2.3% had good nutritional literacy, as shown in Figure 1. Table 3 provides a summary of each individual nutrition literacy instrument item responses. Out of the 265 participants, 84.2% (*n* = 223) participants said they knew eating rice or chapatti or cereal gives them energy, whereas 9.4% (*n* = 25) said they did not know. Majority of the participants (80%), said they knew fruits and vegetables prevented them from diseases, however, 4.9% said that they do not think so. About 148 (55.8%) respondents said that they do not think breastfeeding is the best option for newborns, and only 34 (12.8%) agreed that breast milk is the best choice for newborn babies. Majority of the study respondents (52.1%) said that they do not know how to read a food label, whereas only 17.7% said they can read a food label. 159 (60%) of the respondents said they cannot search for authentic nutrition information on the internet, whereas 34 (12.8%) believed the nutrition information present on the internet is authentic.

**Table 3:** Nutrition literacy instrument responses.

Item	Yes		No		Do Not Know	
	Frequency	Percentage (%)	Frequency	Percentage (%)	Frequency	Percentage (%)
I know that eating chapati/ rice/ cereal is important because it gives me energy.	223	84.2	17	06.4	25	09.4
I understand that eating meat/eggs/lentils is essential for my health.	207	78.1	18	6.8	40	15.1
I realize that adequate consumption of milk and/or milk products is associated with strong bones.	149	56.2	70	26.4	46	17.4
I know that regular consumption of fresh fruits and vegetables can prevent me from several diseases.	212	80.0	13	04.9	40	15.1
I understand that too much consumption of salt and sugar is bad for health.	93	35.1	116	43.8	56	21.1
I know that eating meat is required for maintaining appropriate iron levels in blood.	67	25.3	94	35.5	104	39.2
I understand that sunlight is necessary to get adequate vitamin D.	78	29.4	66	24.9	121	45.7
I know that exclusive breastfeeding is the best choice for newborns.	34	12.8	148	55.8	83	31.3
I realize that mental stress negatively affects diet.	26	09.8	119	44.9	120	45.3
I know that a healthy diet positively influences sleep.	48	18.1	85	32.1	132	49.8
I am aware that washing hands with soap before handling food prevents several diseases.	162	61.1	37	14.0	66	24.9
I can read a food label.	47	17.7	138	52.1	80	30.2
I realize that one should always consult a healthcare provider before taking any nutritional supplement.	78	29.4	116	43.8	71	26.8
I can search for authentic nutrition related information on the internet.	34	12.8	159	60.0	72	27.2

### 3.3 Association between nutritional literacy and socio-demographic factors

Nutritional literacy was found to be statistically significantly associated with three of the socio-demographic variables; gender (*p* = 0.024), age group (*p* = 0.049) and education (*p* < 0.001), as shown in Table 4.

**Table 4:** Association between nutritional literacy and socio-demographic factors.

Variable	Categories	Nutritional literacy		*p*-value
		Poor N (%)	Moderate/Good N (%)	
Gender	Male	127 (53.8)	22 (75.9)	0.024*
	Female	109 (46.2)	07 (24.1)	
Age group	≤30 years	67 (28.4)	14 (48.3)	0.049*
	31–45 years	73 (30.9)	09 (31.0)	
	>45 years	96 (40.7)	06 (20.7)	
Education	Illiterate	144 (61)	03 (10.3)	<0.001*
	Primary	55 (23.3)	03 (10.3)	
	Middle	10 (4.2)	04 (13.8)	
	Matric	18 (5.5)	05 (17.2)	
	Inter	08 (3.4)	06 (20.7)	
	Graduation or above	06 (2.5)	08 (27.6)	
Employment status	Barber	03 (1.3)	00 (0.0)	0.923
	Beautician	04 (1.7)	00 (0.0)	
	Businessman	04 (1.7)	00 (0.0)	
	Clerk	09 (3.8)	00 (0.0)	
	Computer Operator	01 (0.4)	00 (0.0)	
	Cook	09 (3.8)	00 (0.0)	
	Dishwasher	03 (1.3)	00 (0.0)	
	Driver	05 (2.1)	00 (0.0)	
	Gardener	06 (2.5)	02 (6.9)	
	Laboratory Attendant	03 (1.3)	00 (0.0)	
	Laborer	24 (10.2)	01 (3.4)	
	Lecturer	01 (0.4)	00 (0.0)	
	Maid	28 (11.9)	06 (20.7)	
	Mason	15 (6.4)	03 (10.3)	
	Un-employed	39 (16.5)	06 (20.7)	
	Nurse	07 (3.0)	00 (0.0)	
	Office Boy	07 (3.0)	03 (10.3)	
	Painter	06 (2.5)	01 (3.4)	
	Peon	05 (2.1)	00 (0.0)	
	Postman	02 (0.8)	00 (0.0)	
	Salesman	04 (1.7)	00 (0.0)	
	Security Guard	05 (2.1)	01 (3.4)	
	Shopkeeper	11 (4.7)	01 (3.4)	
	Sweeper	21 (8.9)	05 (17.2)	
	Tailor	07 (3.0)	00 (0.0)	
	Teacher	04 (1.7)	00 (0.0)	
	Waiter	02 (0.8)	00 (0.0)	
	Welder	01 (0.4)	00 (0.0)	
Marital status	Single	76 (32.2)	05 (17.2)	0.099
	Married	160 (67.8)	24 (82.8)	
Family structure	Independent	24 (10.2)	03 (10.3)	1.000
	Joint	212 (89.8)	26 (89.7)	

*p < 0.05 was considered statistically significant.

### 3.4 Odds for poor nutritional literacy with socio-demographic factors

In comparison to female participants, male participants were less likely to have poor nutritional literacy (OR = 0.371, *p* = 0.029) as shown in Table 5. Compared to participants over 45, participants under 30 years old (OR = 0.299, *p* = 0.019) and those between 31 and 45 years old (OR = 0.507, *p* = 0.216) are less likely to have poor nutritional literacy. Illiterate and individuals with primary education were at a significantly higher risk of poor nutrition literacy as compared to those with a graduate degree or higher education (*p* < 0.001). Employment status, marital status, and family structure did not show any significant relationship with poor nutrition literacy (*p* > 0.05).

**Table 5:** Odds for poor nutritional literacy with socio-demographic factors.

Variable	Categories	*p*-value	OR (95% CI)
Gender	Male	0.029*	0.371 (0.153–0.901)
	Female		1.000
Age Group	≤30 years	0.019*	0.299 (0.109–0.818)
	31–45 years	0.216	0.507 (0.173–1.488)
	>45 years		1.000
Education	Illiterate	<0.001*	64.000 (13.475–303.978)
	Primary	<0.001*	24.444 (5.076–117.715)
	Middle	0.133	3.333 (0.693–16.022)
	Matric	0.099	3.467 (0.791–15.197)
	Inter	0.451	1.778 (0.398–7.943)
	Graduation or above		1.000
Employment Status	Un-employed	0.361	0.361 (0.068–1.905)
	Private Job	0.252	0.417 (0.093–1.864)
	Govt. Job	0.699	0.611 (0.050–7.397)
	Own business		1.000
Marital Status	Single	0.107	2.280 (0.838–6.207)
	Married		1.000
Family Structure	Independent	0.977	0.981 (0.276–3.485)
	Joint		1.000

*p < 0.05 was considered statistically significant.

### 3.5 Association between nutritional literacy and health assessment

There was no significant association between nutritional literacy and anthropometric/clinical factors showed that body mass index (BMI), waist circumference, nausea, and stool frequency status (*p* > 0.05) as shown in Table 6. Of the participants falling in the poor nutrition category, 5.9% were underweight, 53% had normal BMI, 9.3% were overweight, and 31.8% were obese, with no significant differences compared to those with moderate/good literacy (*p* = 0.228). Similarly, waist circumference (*p* = 0.772), nausea (*p* = 1.000), and stool frequency (*p* = 1.000) were not significantly associated with nutrition literacy levels. However, skin color was significantly associated with nutritional literacy (*p* = 0.030), as a higher proportion of participants with cyanosis (13.8%) and jaundice (6.9%) had moderate/good nutritional literacy compared to those with poor literacy.

**Table 6:** Association of poor nutritional literacy with BMI, waist circumference, skin color, nausea and stool frequency.

Variables	Categories	Nutritional literacy		*p*-value
		Poor N (%)	Moderate/Good N (%)	
Body mass index	Underweight	14 (5.9)	01 (3.4)	0.228
	Normal	125 (53)	16 (55.2)	
	Overweight	22 (9.3)	06 (20.7)	
	Obese	75 (31.8)	06 (20.7)	
Waist circumference	High	46 (19.5)	05 (17.2)	0.772
	Normal	190 (80.5)	24 (82.8)	
Skin color	Pallor(Pale)	15 (6.4)	02 (6.9)	0.030*
	Jaundice(Yellow)	06 (2.5)	02 (6.9)	
	Cyanosis(Blue)	08 (3.4)	04 (13.8)	
	Normal	207 (87.7)	21 (72.4)	
Nausea	Yes	06 (2.5)	00 (0.0)	1.000
	No	230 (97.5)	29 (100)	
Stool frequency	Loose	04 (1.7)	00 (0.0)	1.000
	Constipated	25 (10.6)	03 (10.3)	
	Normal	207 (87.7)	26 (89.7)	

*p < 0.05 was considered statistically significant.

### 3.6 Odds of poor nutritional literacy with health assessment

Results reported in Table 7 show that compared to obese participants, those who were overweight had lower odds of poor nutritional literacy (OR = 0.293, *p* = 0.050). Waist circumference was not significantly associated with nutrition literacy (*p* = 0.772). Among clinical signs, cyanosis was significantly associated with nutritional literacy (OR = 0.203, *p* = 0.015), indicating lower odds of poor nutritional literacy among participants with cyanosis compared to those with normal skin color.

**Table 7:** Odds for poor nutritional literacy with BMI, waist circumference, skin color, nausea and stool frequency.

Variables	Categories	Nutritional literacy	
		*p*-value	OR(95% CI)
Body mass index	Underweight	0.919	1.120 (0.125–10.033)
	Normal	0.348	0.625 (0.234–1.667)
	Overweight	0.050*	0.293 (0.086–1.001)
	Obese		1.000
Waist circumference	High	0.772	1.162 (0.421–3.210)
	Normal		1.000
Skin color	Pallor (Pale)	0.728	0.761 (0.163–3.557)
	Jaundice (Yellow)	0.161	0.304 (0.058–1.604)
	Cyanosis (Blue)	0.015*	0.203 (0.056–0.731)
	Normal		1.000
Nausea	Yes	0.999	NA
	No		1.000
Stool frequency	Loose	0.999	NA
	Constipated	0.944	1.047(0.295–3.709)
	Normal		1.000

*p < 0.05 was considered statistically significant.

### 3.7 Association between nutritional literacy and vital signs

Our study found no significant associations between nutritional literacy and any of the recorded vital signs (*p* > 0.05) as shown in Table 8. Among participants with poor nutritional literacy, 1.7% had hyperthermia, 14.8% had hypertension, 25.8% were pre-diabetic, and 8.5% were diabetic.

**Table 8:** Association of nutritional literacy with vital signs (body temperature, blood pressure, pulse/heart rate, breathing/respiratory rate, and blood sugar random).

Variables	Categories	Nutritional literacy		*p*-value
		Poor N (%)	Moderate/Good N (%)	
Body temperature	Hyperthermia (>99)	04 (1.7)	00 (0.0)	1.000
	Normal	232 (98.3)	29 (100)	
Blood pressure	Hypertension	35 (14.8)	04 (13.8)	1.000
	Normal	201 (85.2)	25 (86.2)	
Pulse/heart rate	Bradycardia (<60)	02 (0.8)	00 (0.0)	1.000
	Tachycardia (>100)	01 (0.4)	0 (0.0)	
	Normal (60–100)	233 (98.7)	29 (100)	
Breathing/respiratory rate	Bradypnea (<12)	02 (0.8)	00 (0.0)	0.448
	Tachypnea (>20)	08 (3.4)	02 (6.9)	
	Normal (12–20)	226 (95.8)	27 (93.1)	
Blood sugar random	Pre-diabetes	61 (25.8)	06 (20.7)	0.779
	Diabetes	20 (8.5)	03 (10.3)	
	Normal	155 (65.7)	20 (69)	

*p* < 0.05 was considered statistically significant.

### 3.8 Odds of poor nutritional literacy with vital signs

Table 9 shows there was no significant association between nutritional literacy and body temperature, blood pressure, pulse rate, respiratory rate, or blood sugar levels (*p* > 0.05). Specifically, participants with hypertension, pre-diabetes, or had odds similar to those with normal parameters. Body temperature and pulse rate was also not significantly associated with nutrition literacy (*p* > 0.05).

**Table 9:** Odds of poor nutritional literacy with vital signs (body temperature, blood pressure, pulse/heart rate, breathing/respiratory rate, and blood sugar random).

Variables	*p*-value	OR(95% CI)
Body temperature		
Hyperthermia (>99)	0.999	NA
Normal		1.000
Blood pressure		
Hypertension	0.882	1.088 (0.357–3.318)
Normal		1.000
Pulse/heart rate		
Bradycardia (<60)	0.999	NA
Tachycardia (>100)	1.000	NA
Normal (60–100)		1.000
Breathing/respiratory rate		
Bradypnea (<12)	0.999	NA
Tachypnea (>20)	0.366	0.478 (0.096–2.367)
Normal (12–20)		1.000
Blood sugar random		
Pre-diabetes	0.579	1.312 (0.503–3.423)
Diabetes	0.820	0.860 (0.234–3.156)
Normal		1.000

*p* < 0.05 was considered statistically significant.

## 4 Discussion

This study aimed to assess the nutritional literacy levels of adults and their associations with various socio-demographic variables within a marginalized community of Islamabad. Lack of nutrition education and awareness in adults is associated with poor disease management, and overall compromised health status (23). Establishing an understanding of how the different health dimensions are influenced by nutritional literacy can improve population health outcomes.

The first aim of the study was to assess the level of nutrition literacy among marginalized community members and reported an 89.1% prevalence of poor nutritional literacy levels. High and moderate nutrition literacy was only reported in 2.3 and 8.7% of the participants. The results highlight a significant gap in nutrition education and awareness, particularly in the vulnerable population groups. A study in Lahore reported that 65.3% of the study participants had high nutrition knowledge, while 34.7% reported low nutrition literacy scores. However, half of the participants reporting high nutrition literacy were unaware on how to read nutrition labels. Online resources was the most reported source of acquiring nutrition information (12). The higher level of reported nutrition literacy in this study can be attributed to the study population being primarily urban residents with overall higher education status, as compared to our study sample of marginalized individuals. These findings further underscore how social determinants such as education, household income, and societal disparities may negatively impact nutrition literacy. Therefore, these inequities must be addressed through upstream, approaches focusing on equitable education, accessible healthcare, and community based nutrition interventions to improve health literacy in resource constrained settings.

Gender was found to be significantly associated with nutritional literacy (*p* = 0.029), and the odds of having poor nutrition literacy were less in females (OR = 0.371, *p* = 0.029). A significant association (*p* = 0.04) between both variables was also reported in another study, while also reporting that males were more likely to have higher odds of poor nutrition literacy as compared to females (OR = 0.64), which coincides with our study’s findings (29). Similar findings were reported by a Turkish study which found higher nutrition literacy scores for females as compared to males (*p* < 0.001). The study attributed these results to women’s role as the primary provider of family meals in the household. As a result, they are able to acquire more nutritional knowledge and awareness from their peers and other information sources (30). Therefore, incorporating nutrition education in gender specific policies and programs, can promote nutrition literacy among females. Strategies like adult literacy initiatives, community out-reach programs, and women specific projects, can all help promote women as drivers of dietary change in household and the community.

Age group of the participants was also significantly associated with nutrition literacy (*p* = 0.019), in our study. Multiple regression analysis found that the younger aged individuals (45 years and under) were less likely to report poor nutrition literacy scores (OR = 0.507, *p* = 0.216). These results are similar to a study in Greece, which reported a significant decline in nutrition literacy as age progresses (*p* < 0.001). This can be linked to the age related cognitive impairment, which makes it difficult to attain, analyze, and implement new information. Older adults diagnosed with dementia have worse nutrition literacy scores, however, even in the absence of a dementia diagnosis, a negative association between increased age and nutrition literacy has been observed (31). Similarly, a Chinese study also reported a significant association between an individual’s age and their nutrition literacy (*p* < 0.001). The study found that nutrition literacy scores dropped as individuals aged, and suggested that the declining cognitive function amplifies the nutrition knowledge gap in geriatrics. This is further associated with decreased nutrient intakes and compromised nutritional status in the older adults, as they lack nutrition awareness (32). These findings highlight the need for age-sensitive literacy policies, as the geriatric population forms a vulnerable group with limited comprehension of nutrition and healthcare information. Integrating community health programs with age and culturally modified nutrition guidance, can help promote nutrition literacy in this population group.

In our study, education levels and nutrition literacy scores were also significantly associated (*p* < 0.001), and a lack of higher education was linked with higher odds of being nutritionally illiterate (OR = 64.000, *p* < 0.001). Chinese adults with formal college education were reported to have higher nutrition literacy scores (OR = 4.465, *p* < 0.0001), and the study also reported significant associations between education level and nutrition knowledge in the population (*p* < 0.0001). The behavior change theory can be used to explain the significant association between these two variables as education attainment positively influences behavior modification. With higher level of formal education, the nutrition awareness also increases, thereby fostering nutrition literacy and changes in food related attitudes and practices (33). Furthermore, the Human Capital Theory (HCT) states that education is an investment into human health and productivity, and provides individuals with the skills needed to change their health and nutrition goals (34). Tertiary and higher level education is associated with higher levels of self-reported health, improved nutritional well-being, and decreased morbidity and mortality risks. Education is particularly important in improving nutrition and health indicators in marginalized communities (35).

The mean weight reported for our study sample was 60.96 ± 9.71 kg, and mean BMI was 23.22 ± 4.30 kg/m2. Of the 265 study participants, 53.2% had a normal BMI, while 10.6% were overweight. These findings are similar to another study which reported the mean BMI as 22.91 kg/m^2^. However, it reported 22.8% of the participants to be overweight, which is much higher than our findings of 10.6% (36). This can be attributed to our study participants belonging to a marginalized community with limited financial resources to engage in unhealthy lifestyle practices such as eating out, associated with various behavioral risk factors. The relationship between BMI and nutritional literacy level was however, non-significant (*p* = 0.228) in our study. A study in Lebanon explored the relationship between BMI and nutritional literacy but also reported a non-significant relationship between the two variables (*p* = 0.796). This lack of relationship stems from the fact that merely theoretical knowledge is not enough to develop behavior change or lifestyle modifications suggests that theoretical health and nutrition knowledge must be coupled with behavior change competences, and support frameworks to produce significant body measurement changes (37).

Of the 265 participants, 19.2% of the participants had a high waist circumference, whereas 80.8% were normal. Waist circumference was not significantly associated with nutritional literacy (*p* = 0.772). The results are similar to a Turkish study also found a non-significant relationship between waist circumference and health literacy (*p* = 0.060) (38). However, a Chinese study found conflicting results where a significant relationship between health literacy and waist circumference was reported (*p* < 0.001). Lower nutrition literacy was linked with higher waist circumference. This is attributed to the causal relationship between lower health literacy levels and incidence of behavioral risk factors. Modifiable risk factors such waist circumference, are strongly tied to lack of general health and dietary awareness. People with lower health literacy are more likely to exhibit poor lifestyles behaviors and are more prone to clustering to risk factors (39).

Our study also assessed the prevalence of hypertension and diabetes in the study population and found a 14.8 and 8.5% prevalence of both health conditions, respectively. Additionally, hypertensive participants had more odds of reporting poor nutritional literacy (OR = 1.088, *p* = 0.882). Another study also found a significant relationship between health literacy score and glycemic control in diabetics (*p* < 0.001) and reported only 23.5% respondents had adequate health literacy. Health and nutritional literacy promotes participants” comprehension and disease management skills. Poor nutrition literacy results in lack of understanding dietary guidance resulting in worsening of disease. Good health and nutritional literacy aids compliance to medical, dietary, and physical activity advice, and prevents development of various diseases (40).

Our study also found a significant association between cyanosis and nutritional literacy, with cyanotic participants showing significantly lower odds of poor nutritional literacy (OR = 0.203, *p* = 0.015). This can be explained by the fact that individuals exhibiting skin changes are more likely to get healthcare advice and awareness regarding their condition. This improves their odds of earlier diagnosis and treating the underlying health condition (41). However, further research is needed to explore if this relationship is causal or confounded by other existing health conditions. Overall, the findings indicate the need for multi-sectoral policies that tackle the underlying social and structural determinants of nutrition literacy. Resource constrained setting, as in LMICs, require upstream approaches, involving the community, for system and policy reforms that empower individuals to make informed dietary choices. Current programs running in the country, such as the lady health worker program, can integrate education and familiarity to digital learning tools, to address barriers to formal education.

The current study has certain limitations that need to be addressed. Firstly the study was conducted in a marginalized and underserved community, which may limit the generalizability of the findings. These results may not be applicable to more broader or diverse population groups. The high prevalence of poor nutrition literacy may be attributed to underlying socio-demographic discrepancies in education, gender, and age. Therefore, the reported results should not be perceived as a reflection of the general population. Secondly, the study included participants from only 2 out of the 8 recognized marginalized communities, due to limited resources and funding. While purposive sampling was adequate in providing access to the population, it introduced selection bias by restricting the representativeness of the results. We aim to scale up the study in the future, to enhance generalizability and ensure comprehensive understanding of nutrition literacy across varying population groups. Thirdly, incorporating ‘I do not know’ as a response option and scoring it as 0 may overestimate the prevalence of low nutrition literacy. While this approach is consistent with prior nutrition literacy studies, as cited above, future research may benefit from considering and analyzing ‘I do not know’ responses separately, in order to not overstate the burden of nutrition illiteracy in a population. Additionally, the study did not include a comparative analysis for rural urban settings, due to funding and resource constraints, which may restrict representativeness of the findings. Future research is however, planned to include a more diverse sample, allowing for comparisons across different population groups, thereby enhancing representativeness. The analysis was based on univariate analysis, and does not account for potential confounders, such as age, gender, and educational background. Subsequent studies will utilize multivariate and sensitivity analysis to ensure robustness of results. It should also be noted that observations for certain variables such as cyanosis may arise from random variation or confounding factors, which were not accounted for in the analysis. Nonetheless, the findings highlight important gaps in nutrition literacy within marginalized and vulnerable population groups. They further underscore the need for contextually appropriate nutrition interventions at the community level, particularly in underserved populations.

## 5 Conclusion

Nutritional literacy is vital for shaping the food, nutrition, and health behaviors of population groups, particularly those living in disadvantaged conditions. It is now gaining recognition as a main component of health literacy, and as an enhancer of both health and nutrition outcomes. While planning and implementing nutrition literacy interventions, it is important to incorporate the socio-demographic and health factors that influence nutrition literacy status. This can help promote nutrition literacy as a preventive measure against several chronic diseases, especially in resource poor settings. Our study found that the adult residents of marginalized community living in Islamabad had poor nutritional literacy. Enhancing nutritional literacy levels in marginalized communities requires a multi-level, comprehensive approach. This includes targeting interventions at the individual, interpersonal, organizational, community, and policy levels. Tailoring recommendations for each level can help efficiently and effectively target the root causes of nutritional illiteracy.

## Data Availability

The raw data supporting the conclusions of this article will be made available by the authors, without undue reservation.
