# Supplementary material for: Prevalence of nutritional literacy and associated factors among adult residents: a cross-sectional study from marginalized Community in Islamabad, Pakistan
Source: Front Public Health. 2025 Nov 25;13:1698201. doi: 10.3389/fpubh.2025.1698201 (PMC12687912; doi:10.3389/fpubh.2025.1698201)
Supplement: Supplementary file 1 [file Table_1.docx]

**Supplementary File A**

**Nutritional Literacy Questionnaire**

**Gender:** Male/Female

**Age:**

**Education:**

**Occupation:**

**Marital Status:** Single/Married

**Family Structure:** Nuclear/Joint

**Nutritional Literacy Questionnaire**

| **S. No.** | **Item** | **Yes** | **No** | **Do not know** |
| --- | --- | --- | --- | --- |
| 1 | I know that eating chapati/ rice/ cereal is important because it gives me energy. |  |  |  |
| 2 | I understand that eating meat/eggs/lentils is essential for my health. |  |  |  |
| 3 | I realize that adequate consumption of milk and/or milk products is associated with strong bones. |  |  |  |
| 4 | I know that regular consumption of fresh fruits and vegetables can prevent me from several diseases. |  |  |  |
| 5 | I understand that too much consumption of salt and sugar is bad for health. |  |  |  |
| 6 | I know that eating meat is required for maintaining appropriate iron levels in blood. |  |  |  |
| 7 | I understand that sunlight is necessary to get adequate vitamin D. |  |  |  |
| 8 | I know that exclusive breastfeeding is the best choice for newborns. |  |  |  |
| 9 | I realize that stress negatively affects diet. |  |  |  |
| 10 | I know that a healthy diet positively influences sleep. |  |  |  |
| 11 | I am aware that washing hands with soap before handling food prevents several diseases. |  |  |  |
| 12 | I can read a food label. |  |  |  |
| 13 | I realize that one should always consult a healthcare provider before taking any nutritional supplement. |  |  |  |
| 14 | I can search for authentic nutrition related information on the internet. |  |  |  |

**Obtained Score =**

**Total score = 14**
