# Supplementary material for: Prevalence of nutritional literacy and associated factors among adult residents: a cross-sectional study from marginalized Community in Islamabad, Pakistan
Source: Front Public Health. 2025 Nov 25;13:1698201. doi: 10.3389/fpubh.2025.1698201 (PMC12687912; doi:10.3389/fpubh.2025.1698201)
Supplement: Supplementary file 2 [file Table_2.docx]

**Supplementary File B**

**Table S1: Content Validity Index for Clarity of Nutritional Literacy Research Instrument**

| **Item** | **Description** | **Exp. 1** | **Exp. 2** | **Exp. 3** | **Exp. 4** | **Exp. 5** | **Exp. 6** | **CVI** |
| --- | --- | --- | --- | --- | --- | --- | --- | --- |
| 1 | I am aware of the concept of a balanced diet. | 4 | 4 | 1 | 2 | 4 | 4 | 0.67 |
| 2 | I know that eating chapati/ rice/ cereal is important because it gives me energy. | 4 | 4 | 4 | 4 | 4 | 4 | 1.00 |
| 3 | I understand that eating meat/eggs/lentils is essential for my health. | 4 | 4 | 4 | 4 | 4 | 4 | 1.00 |
| 4 | I realize that adequate consumption of milk and/or milk products is associated with strong bones. | 4 | 4 | 4 | 4 | 4 | 4 | 1.00 |
| 5 | I know that regular consumption of fresh fruits and vegetables can prevent me from several diseases. | 4 | 4 | 4 | 4 | 4 | 4 | 1.00 |
| 6 | I understand that too much consumption of salt and sugar is bad for health. | 3 | 4 | 4 | 3 | 4 | 3 | 1.00 |
| 7 | I know that eating meat is required for maintaining appropriate iron levels in blood. | 4 | 4 | 4 | 4 | 4 | 4 | 1.00 |
| 8 | I understand that sunlight is necessary to get adequate vitamin D. | 4 | 4 | 4 | 4 | 4 | 4 | 1.00 |
| 9 | I know that exclusive breastfeeding is the best choice for newborns. | 4 | 4 | 4 | 4 | 4 | 4 | 1.00 |
| 10 | I realize that mental stress negatively affects diet. | 4 | 3 | 4 | 4 | 3 | 4 | 1.00 |
| 11 | I know that a healthy diet positively influences sleep. | 4 | 3 | 4 | 4 | 3 | 4 | 1.00 |
| 12 | I am aware that washing hands with soap before handling food prevents several diseases. | 4 | 4 | 4 | 4 | 4 | 4 | 1.00 |
| 13 | I can read a food label. | 4 | 4 | 4 | 4 | 4 | 4 | 1.00 |
| 14 | I realize that one should always consult a healthcare provider before taking any nutritional supplement. | 4 | 3 | 4 | 4 | 3 | 4 | 1.00 |
| 15 | I can search for authentic nutrition related information on the internet. | 4 | 4 | 4 | 4 | 4 | 4 | 1.00 |

1=Not clear, 2=Item needs some revision, 3=Clear but minor revision is required, 4=Very clear

**Table S2: Content Validity Index for Relevance of Nutritional Literacy Research Instrument**

| **Item** | **Description** | **Exp. 1** | **Exp. 2** | **Exp. 3** | **Exp. 4** | **Exp. 5** | **Exp. 6** | **CVI** |
| --- | --- | --- | --- | --- | --- | --- | --- | --- |
| 1 | I am aware of the concept of a balanced diet. | 4 | 1 | 2 | 4 | 4 | 4 | 0.67 |
| 2 | I know that eating chapati/ rice/ cereal is important because it gives me energy. | 4 | 4 | 4 | 4 | 4 | 2 | 0.83 |
| 3 | I understand that eating meat/eggs/lentils is essential for my health. | 4 | 4 | 3 | 4 | 3 | 4 | 1.00 |
| 4 | I realize that adequate consumption of milk and/or milk products is associated with strong bones. | 4 | 4 | 4 | 4 | 4 | 4 | 1.00 |
| 5 | I know that regular consumption of fresh fruits and vegetables can prevent me from several diseases. | 4 | 4 | 4 | 4 | 4 | 4 | 1.00 |
| 6 | I understand that too much consumption of salt and sugar is bad for health. | 4 | 4 | 4 | 4 | 2 | 4 | 0.83 |
| 7 | I know that eating meat is required for maintaining appropriate iron levels in blood. | 4 | 4 | 4 | 4 | 4 | 4 | 1.00 |
| 8 | I understand that sunlight is necessary to get adequate vitamin D. | 4 | 1 | 3 | 4 | 4 | 4 | 0.83 |
| 9 | I know that exclusive breastfeeding is the best choice for newborns. | 4 | 4 | 4 | 4 | 4 | 4 | 1.00 |
| 10 | I realize that mental stress negatively affects diet. | 3 | 4 | 4 | 3 | 4 | 3 | 1.00 |
| 11 | I know that a healthy diet positively influences sleep. | 3 | 4 | 3 | 3 | 4 | 3 | 1.00 |
| 12 | I am aware that washing hands with soap before handling food prevents several diseases. | 4 | 3 | 4 | 2 | 4 | 4 | 0.83 |
| 13 | I can read a food label. | 4 | 2 | 4 | 4 | 4 | 4 | 0.83 |
| 14 | I realize that one should always consult a healthcare provider before taking any nutritional supplement. | 4 | 4 | 4 | 4 | 4 | 4 | 1.00 |
| 15 | I can search for authentic nutrition related information on the internet. | 4 | 4 | 4 | 4 | 2 | 4 | 0.83 |

1=Not relevant, 2=Item needs some revision, 3=Relevant but minor revision is required, 4=Very relevant

**Table S3: Content Validity Index for Simplicity of Nutritional Literacy Research Instrument**

| **Item** | **Description** | **Exp. 1** | **Exp. 2** | **Exp. 3** | **Exp. 4** | **Exp. 5** | **Exp. 6** | **CVI** |
| --- | --- | --- | --- | --- | --- | --- | --- | --- |
| 1 | I am aware of the concept of a balanced diet. | 4 | 3 | 1 | 4 | 1 | 2 | 0.50 |
| 2 | I know that eating chapati/ rice/ cereal is important because it gives me energy. | 4 | 4 | 4 | 4 | 4 | 4 | 1.00 |
| 3 | I understand that eating meat/eggs/lentils is essential for my health. | 3 | 4 | 4 | 3 | 4 | 3 | 1.00 |
| 4 | I realize that adequate consumption of milk and/or milk products is associated with strong bones. | 4 | 4 | 4 | 4 | 4 | 4 | 1.00 |
| 5 | I know that regular consumption of fresh fruits and vegetables can prevent me from several diseases. | 4 | 4 | 4 | 4 | 4 | 4 | 1.00 |
| 6 | I understand that too much consumption of salt and sugar is bad for health. | 4 | 4 | 4 | 4 | 4 | 4 | 1.00 |
| 7 | I know that eating meat is required for maintaining appropriate iron levels in blood. | 3 | 3 | 4 | 3 | 3 | 3 | 1.00 |
| 8 | I understand that sunlight is necessary to get adequate vitamin D. | 4 | 4 | 4 | 4 | 4 | 4 | 1.00 |
| 9 | I know that exclusive breastfeeding is the best choice for newborns. | 4 | 4 | 4 | 4 | 4 | 4 | 1.00 |
| 10 | I realize that mental stress negatively affects diet. | 3 | 3 | 4 | 3 | 3 | 3 | 1.00 |
| 11 | I know that a healthy diet positively influences sleep. | 4 | 3 | 4 | 4 | 3 | 4 | 1.00 |
| 12 | I am aware that washing hands with soap before handling food prevents several diseases. | 4 | 4 | 4 | 4 | 4 | 4 | 1.00 |
| 13 | I can read a food label. | 4 | 4 | 4 | 4 | 4 | 4 | 1.00 |
| 14 | I realize that one should always consult a healthcare provider before taking any nutritional supplement. | 4 | 4 | 4 | 4 | 4 | 4 | 1.00 |
| 15 | I can search for authentic nutrition related information on the internet. | 4 | 4 | 4 | 4 | 4 | 4 | 1.00 |

1=Not Simple, 2=Item needs some revision, 3=Simple but minor revision is required, 4=Very simple
